# Metallurgy, Properties and Applications of Superaustenitic Stainless Steels—SASSs

**DOI:** 10.3390/ma18133079

**Published:** 2025-06-28

**Authors:** Alessio Malandruccolo, Cinzia Menapace, Igor Giroletti

**Affiliations:** 1 Department of Industrial Engineering—DII, University of Trento, 38123 Trento, Italy; cinzia.menapace@unitn.it; 2 Omeco Srl, 20900 Monza, Italy; igor.giroletti@omecosrl.it

**Keywords:** superaustenitic stainless steels, precipitates, microstructure, mechanical properties, corrosion resistance, processing

## Abstract

Superaustenitic stainless steels (SASSs) are one of the families of high-performance stainless steels, the so-called “super” grades. While sharing the face-centered cubic lattice structure typical of standard austenitic stainless steels, their chemical composition is significantly more complex. This enables them to offer an exceptional balance of superior corrosion resistance and high mechanical strength. However, the intricate chemical makeup of SASSs brings challenges, such as the phenomenon of segregation and precipitation of deleterious intermetallics. Consequently, this leads to several challenges in their processing and use. This work aims to present SASSs in detail, starting from their chemistry and metallurgy and ending with processing and applications. Hence, the first part will be dedicated to the analysis of chemistry, resulting grades, microstructure and secondary phases along with the conditions determining their formation. Afterwards, physical, mechanical and corrosion resistance characteristics will be set forth in such a way as to understand their origin and implications for processing and possible uses, with a focus on processability limitations. In fact, manufacturing and processing options significantly affect the types of products that can be developed, and, when considered alongside material attributes and costs, they help define the target markets for these alloys.

## 1. Introduction

Conventionally, stainless steels are divided into five main families: ferritic, austenitic, martensitic, duplex and precipitation hardening (PH). The differences between them arise from the chemical composition and determine the microstructure and, with particular reference to PH, the strengthening mechanism [1]. Except for the PH family, however, there are additional variants that, because of some of their peculiar characteristics, are referred to as *super* grades. This further sub-classification within individual families also includes superaustenitic stainless steels (SASSs). The prefix “super” is used to emphasize the fact that these steels are part of the high-performance stainless steels group, whose “super performance” compared with standard grades is evident in their enhanced resistance to wet corrosion in its various forms, in particular the localized ones. Indeed, to further emphasize their superior localized corrosion resistance, the conventional criterion for an austenitic stainless steel to be considered part of this group is the attainment of a pitting resistance equivalent (PRE) value of 40 or greater [2,3].

It is well known that the primary driving force behind the development of stainless steels has always been the pursuit of materials with enhanced performance in corrosive environments. This impetus became particularly strong in the first decade of the 20th century, driven by the rapid growth of the cellulose synthesis and oil refining industries. Research and development efforts aimed at formulating steels with increased resistance to chemical agents originated in Germany and subsequently spread across Europe and worldwide, led by top player companies in the metallurgical sector [4]. This has driven the pursuit of extreme performance, particularly in terms of resistance to localized corrosion and stress corrosion cracking (SCC). The interest in developing high-performance alloys with enhanced corrosion resistance along with the aim of achieving improved mechanical properties with similar performance, but lower cost than Ni-based alloys, passed first through the development of the high-performance austenitic AISI 904L and ultimately led to the development of SASSs. In fact, AISI 904L, though frequently included in the SASS group, should instead be considered a precursor to this material family. This is due to both its chemical composition and its PRE value, which does not meet the minimum threshold of 40. In this framework, SASSs represent one of the most recent advancements in austenitic stainless steels [3,5].

SASSs are characterized by a fully stable austenitic microstructure at all temperatures and a chemical composition rich in alloying elements such as Cr, Mo, N and Ni. Achieving this composition became feasible only after the mid-20th century with the introduction of innovative steelmaking technologies, such as the argon oxygen decarburization (AOD) process and, subsequently, vacuum oxygen decarburization (VOD). The development of these materials led to the introduction of several commercial variants, some of which have since been phased out. Currently, SASSs constitute a niche group of alloys. Among these, grade 654SMO is considered to have reached the highest level of performance and alloy development to date. While their market share is a small fraction of the overall stainless steel industry, there is significant interest in these alloys for highly demanding and specialized applications. The processing challenges presented by their chemical composition continue to drive research and development [3].

To present a detailed insight into these materials, this work will provide information about their key chemical and microstructural characteristics. This will serve as the foundation for analyzing their physical, mechanical and corrosion resistance properties. Based on these latter details, considerations will be given regarding the manufacturing technologies employed and the associated processing challenges, with the aim of better understanding the potential applications of these alloys.

## 2. Chemistry and Related Features

The chemical composition of each metal alloy defines its characteristics and, consequently, becomes a key point to understand in order to determine the applicable production processes, the achievable products and the suitable service conditions. Furthermore, the chemical composition also influences the microstructure and the phases that can be formed in the material. In addition, based on the chemical composition, phenomena related to solidification such as segregation can be understood.

### 2.1. Chemical Composition and Microstructure

**Figure 1:**
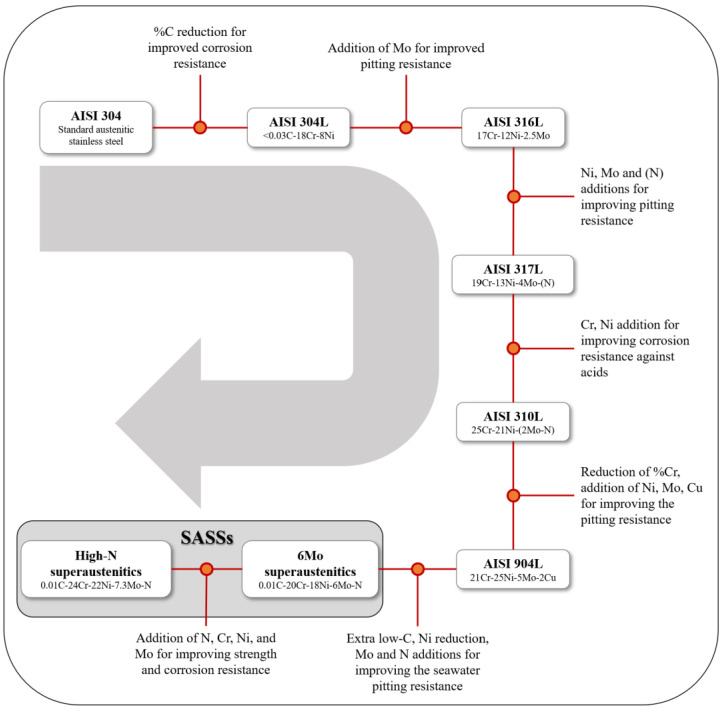
Evolutionary pathway of superaustenitic stainless steels starting from AISI 304. For each member, the key modifications enabling its evolution into the subsequent grade are specified [3,5].

When it comes to austenitic stainless steels, the reference grade when referring to these materials is traditionally AISI 304. As shown in Table 1, AISI 304 has a relatively simple chemical composition with significant amounts of only Cr, Ni and Mn. The evolutionary path that led to the development of SASSs, driven by requirements for increased corrosion resistance, can consider AISI 304 as its starting point, as shown in Figure 1. This process consists essentially of the reduction of C content to very low values and the increase in the amount of specific alloying elements: Ni, Cr, Mo and N. These four key elements have a dual role. On the one hand, they increase the mechanical properties of the steel, and on the other hand, their presence improves corrosion resistance of the material. Ni turns out to be critical in stabilizing the *fcc* matrix at any temperature and improves corrosion resistance through solid-solution hardening. The Ni content in SASSs ranges from 16% to 37%. Cr, on the other hand, has concentrations between 19% and 28% and is the element on which the existence of stainless steels is based; the passive surface layer that protects against corrosion consists mainly of its oxides, and last but not least, Cr also has solid-solution hardening action. Mo has more pronounced mechanical properties—enhancing action through solid-solution hardening, as will be discussed in Section 3, and its content in SASSs ranges between 5% and 8% [3]. Its presence is also particularly beneficial for corrosion resistance because it increases the pitting potential, thus delaying the onset of the phenomenon. Furthermore, Mo additions increase resistance to localized breakdown of the passive film and increases self-healing capacity of the film itself [6]. Both Mo and Cr are α-stabilizing elements, and to counterbalance this action and maintain a stable austenitic matrix, in addition to Ni, N has γ-stabilizing action as well. The N content in SASSs ranges from 0.1% up to the extraordinary value of 0.6%. This high percentage is also a technological challenge given the difficulties associated with maintaining atomic N in solid solution while preventing it from recombining into gaseous N_2_ or reacting with other elements to form nitrides. N also has influence on increasing mechanical properties and promotes resistance against localized corrosion, e.g., pitting, as well [3].

The two most characteristic elements of SASSs are Mo and N. The division of this class of steels into two further groups, as shown in both Figure 1 and Table 1, is based on these elements: 6Mo superaustenitics and high-N superaustenitics. The first group is characterized by an average Mo content of 6% and N content ranging between 0.1% and 0.25%. The 6Mo superaustenitics were initially designed to resist corrosion in seawater and pulp-bleach plants. The high-N superaustenitics were developed at a later stage and, compared with 6Mo superaustenitics, have even higher mechanical properties combined with even better corrosion resistance. They can be classified as one of the latest developments as they entered mass production in the late 20th century. Furthermore, there are few grades in this group. Again, considering Mo and N, their content is as high as 8% for Mo and goes up to an extraordinary 0.6% in the case of N. The development of the high-N grades was driven by the desire to offer a more economical but equally high-performance alternative, in terms of corrosion resistance, to the more expensive Ni alloys for applications with particularly severe service conditions in terms of corrosive environment and stresses to be sustained [3].

**Table 1:** Chemical composition of SASSs alongside standard austenitic AISI 304 and high-performance austenitic AISI 904L. AISI 304 and AISI 904L are shown to provide a comparison on compositional differences Values are expressed in wt% [3,6].

Group	Most Common Name	UNS	C max	Cr	Ni	Mo	Mn	N	Others
Standard austenitics	AISI 304	S30400	0.08	18.0–20.0	8.0–10.5	-	2.0	-	-
High-performance austenitics	AISI 904L	N08904	0.02	19.0–23.0	23.0–28.0	4.0–5.0	2.0	-	Cu = 1.0–2.0
6Mo superaustenitics	20Mo-6	N08026	0.03	22.0–26.0	33.0–37.0	5.0–6.7	-	0.1–0.16	Cu = 2.0–4.0
	NAS 254N	S32053	0.03	23.0	25.0	5.5	-	0.2	-
	25-6Mo	N08926	0.02	19.0–21.0	24.0–26.0	6.0–7.0	1.0	0.15–0.25	Cu = 0.5–1.5
	Uranus SB8	N08932	0.02	24.0–26.0	24.0–26.0	4.7–5.7	-	0.17–0.25	Cu = 1.0–2.0
	254SMO	S31254	0.02	19.5–20.5	17.5–18.5	6.0–6.5	1.0	0.18–0.22	Cu = 0.5–1.0
	Nicrofer 3127 hMo	N08031	0.02	26.0–28.0	30.0–32.0	6.0–7.0	-	0.15–0.25	Cu = 1.0–1.4
	AL-6XN^®^	N08367	0.03	20.0–22.0	23.5–25.5	6.0–7.0	-	0.18–0.25	Cu = 0.75
High-N superaustenitics	Uranus B66	S31266	0.03	23.0–25.0	21.0–24.0	5.0–7.0	3.0	0.35–0.6	Cu = 0.5–3.0 W = 1.0–3.0
	654SMO	S32654	0.02	24.0–26.0	21.0–23.0	7.0–8.0	2.0–4.0	0.45–0.55	Cu = 0.3–0.6
	Nirosta^®^ 4565S	S34565	0.03	23.0–26.0	16.0–19.0	3.5–5.0	3.5–6.5	0.4–0.6	Nb < 0.15

### 2.2. Secondary Phases

**Figure 2:**
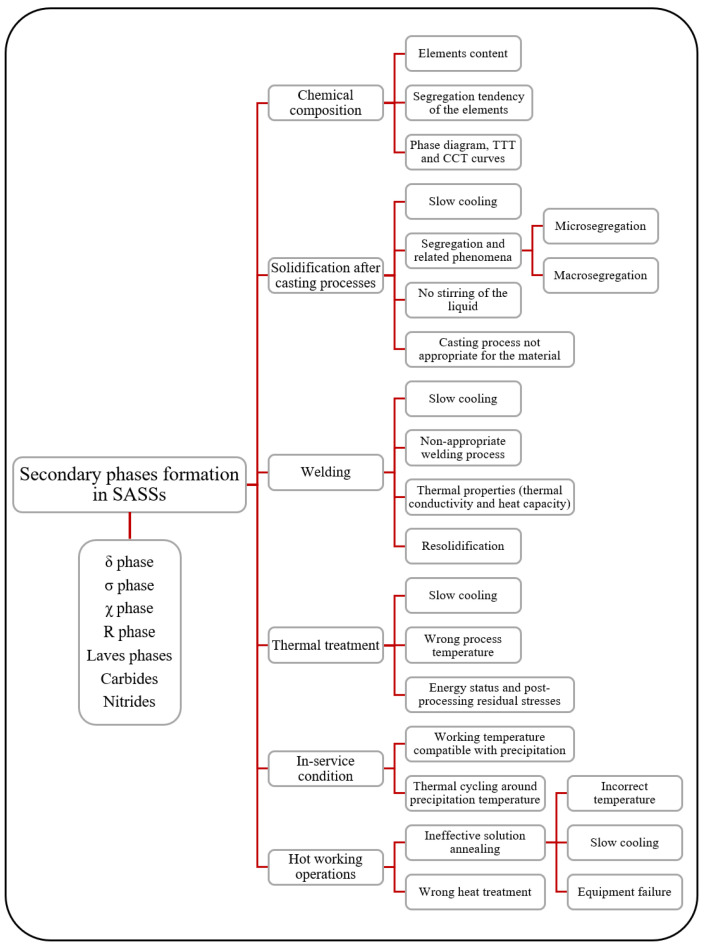
Schematic of the possible conditions that result in the formation of secondary phases in SASSs, along with the list of phases that form most frequently [3,9,10,11,12,13,14].

It is well known that the number of phases that can form and coexist in an alloy is directly proportional to the number of alloying elements and their concentrations. Consequently, it is not surprising that for complex alloys like SASSs, several phases can form in the material, potentially under a variety of circumstances, as summarized in Figure 2. As is evident from Figure 2, the formation of secondary phases is intimately linked not only to the number and concentration of alloying elements within the material but also to their intrinsic segregative tendencies, potentially resulting in micro- or macrosegregation phenomena. For alloys with element-rich compositions, such as SASSs (see Table 1), it is particularly important to identify which elements exhibit a propensity for segregation in the liquid phase during solidification. Among the alloying elements of SASSs, Mo demonstrates the strongest tendency to segregate in the liquid, which is attributed to its partition coefficient significantly greater than 1. Cr also exhibits this tendency, albeit with a considerably lower intensity. This phenomenon is not only favorable for secondary phase formation but is also highly relevant for welding and other solidification-related processes [3].

δ ferrite has *bcc* lattice and can form in SASSs during the solidification process, but due to its decomposition during cooling, it is generally not detected at low temperature. Its presence is generally associated in steels with a worsening of mechanical and corrosion resistance properties. Several studies have investigated the possible modes of δ ferrite formation in SASSs. During solidification, the formation of this phase appears attributable to the segregation of Mo in the interdendritic liquid between the primary austenitic dendrites. The liquid, enriched with a α-stabilizing element, makes the phase thermodynamically stable, allowing it to solidify. Later, at lower temperatures, the δ tends to decompose via a eutectoid reaction, leading to the formation of eutectoid austenite (γ_2_) and σ phase, which presence is deleterious to both mechanical and corrosion resistance properties [7,8]. However, the presence of δ ferrite could be beneficial in welding processes because of its higher solubility for impurities such as P and S. In fully austenitic alloys, the two elements tend to segregate in the liquid during solidification and form low-melting compounds that can lead to cracks along the GBs during solidification or hot working operations. For SASSs, which do not form δ ferrite like other austenitics, control of P and S is therefore paramount to promote the absence of such cracking phenomena [3].

The σ phase is probably the best known and most studied secondary phase because of the deleterious consequences its presence has on material properties. In fact, mechanical and corrosion resistance properties are jeopardized by σ. Corrosion resistance, in particular, is impaired by σ due to matrix depletion of elements that are responsible for the outstanding corrosion resistance of these alloys. The σ phase is a topologically close-packed (TCP) phase characterized by the lack of active slip systems, which, in turn, result in the inherent brittleness of the phase and the material in which it has formed when the volume fraction exceeds a specific threshold value. The σ phase is rich in substitutional elements such as Cr and Mo, both abundant in SASSs. Its formation can occur either due to heating of the solid at the precipitation temperature, as a result of the segregation of Cr and Mo in the liquid during solidification, or as a consequence of eutectoid transformation involving austenite (γ) and decomposition of δ ferrite [7,8]. The latter aspect appears to have been confirmed by experimental evidence obtained with the directional solidification (DS) process, on which rapid cooling was applied to achieve quenching during direction solidification (QDS). This experimental approach enabled a comprehensive characterization of the phenomena occurring in the vicinity of the solidification front, as well as the microstructural evolution of the solid both during cooling and with increasing distance from the solidification front. This consequently highlighted the transformations taking place throughout the process, and a notable outcome of this analysis concerns element segregation both macro and micro [7].

The χ phase is also a TCP phase with a chemical composition similar to that of σ, thus rich in Cr and Mo but with the ability to dissolve C, unlike σ, which is unable to do so. Its precipitation turns out to be easier than that of σ, and this is one of the reasons why its formation occurs earlier in SASSs. In addition to this, it appears that its formation may influence or have some kind of connection with that of σ. In fact, some studies have shown a conversion of Χ to σ for prolonged exposure at precipitation temperatures, as well as preferential nucleation of σ in Mo-rich regions of Χ. Compared with the σ phase, Χ has received less research and experimental effort, and therefore, less dedicated literature is present. In any case, its presence is also associated with deleterious consequences on mechanical and corrosion resistance properties [3].

The R phase belongs to the group of TCP phases and, compared with the two previously mentioned, is detected less frequently in SASSs. Like σ and χ, it is rich in Cr and Mo but with different ratios and seems to form more frequently in regions of the heat-affected zone (HAZ) that have exceeded 800 °C. In some alloys, it is considered a metastable phase. Although its occurrence appears to be less common, it is still associated with a worsening of the properties, particularly due to the depletion of Cr and Mo in the regions surrounding that of its formation [3].

Laves phases detected in SASSs are usually characterized by Fe_2_Mo chemistry and also belong to the family of TCP phases. Like all TCP phases mentioned so far, their presence is not beneficial for mechanical and corrosion resistance properties. Furthermore, given their chemical composition, high Mo contents typical of the best performing SASSs promote the formation of Laves phases. However, experimental evidence reported in the literature seems to indicate that high W contents can also promote their formation [3].

Carbides and nitrides are further secondary phases that can form in SASSs. Given the low C content of these steels (see Table 1), the volume fraction of carbides may not be high. However, for exposure to temperatures in the 700–1100 °C range, the first compounds that form are precisely carbides. The stoichiometries usually found in SASSs are M_23_C_6_ and M_6_C, where M is Cr. Although the presence of carbides does not impair mechanical properties as severely as it happens for TCP phases and indeed can lead to a slight increase in mechanical strength, corrosion resistance is jeopardized due to the depletion of Cr in the areas surrounding the carbides. The presence of nitrides in SASSs, particularly in those grades belonging to the high-N superaustenitics group, is explained precisely by the high N content of these alloys. The formation of nitrides is associated with prolonged exposure to high temperatures, and the main consequence is the change in the chemical composition of the matrix containing them. N depletion has consequences on the corrosion resistance against localized attack and also on mechanical properties, given the particularly pronounced effect of N in this respect [3].

SASSs are supplied in the solution annealed condition, and at most, it is also possible to refer to cold-worked material with a certain degree of work hardening, but still with solution annealed starting condition prior to cold working. Thus, the presence of secondary phases that jeopardize mechanical and corrosion resistance properties can be seen as a failure of the production process, as the solution annealing treatment aims to dissolve the phases in the matrix and obtain a homogeneous material. However, precipitation may also happen due to service conditions that result in temperatures compatible with the formation of such compounds, as determined by the relevant phase diagram. In addition to this, precipitation can also occur as a result of resolidification or heating phenomena associated with welding processes. As shown in Figure 2, phase precipitation can be attributed to six factors, five of which are related to temperature and cooling conditions that promote the phenomenon. The sixth, and most fundamental, is chemical composition, as it determines the possibility of forming secondary phases. The latter, as was partly mentioned earlier, determines the material characteristics and phase diagram to be referred to, as well as the TTT and CCT curves. Thus, chemistry influences the thermodynamics of the material that determines which phase can exist. Additionally, issues related to kinetics are also dependent on chemistry and on the conditions that can be associated with temperature changes over time that promote secondary phase formation. The other five areas in Figure 2 give explanations for the formation of phases as a consequence of temperature-related issues. As can be seen, in all cases, there is either prolonged exposure to temperatures compatible with precipitation or cooling from high temperature slow enough to allow the material to cross the critical temperature ranges and to be exposed to them long enough to form secondary phases [3]. In the case where, as mentioned, the formation of secondary phases is considered a failure in the manufacturing process, the information provided in Figure 2 can be seen as a root-cause analysis associated with precipitation. As a result of this, given a chemical composition that promotes the formation of secondary phases, in order to obtain precipitate-free material, it is necessary to consider appropriate actions to contrast the reported phenomena and conditions.

## 3. Properties of SASSs

Properties of materials are defined as characteristics expressed in terms of a measured and/or measurable response to the application of specific stimuli [15]. They represent key information about the material as they influence the processes to which the material may be subjected, affect its usability, define constraints on service conditions and, consequently, affect the expected useful lifetime of the manufactured products. To give a comprehensive overview of materials such as SASSs, it is essential to analyze some of the physical, mechanical and corrosion resistance properties that most contribute to defining the peculiarities of these materials, starting with the physical ones.

### 3.1. Physical Properties

**Figure 3:**
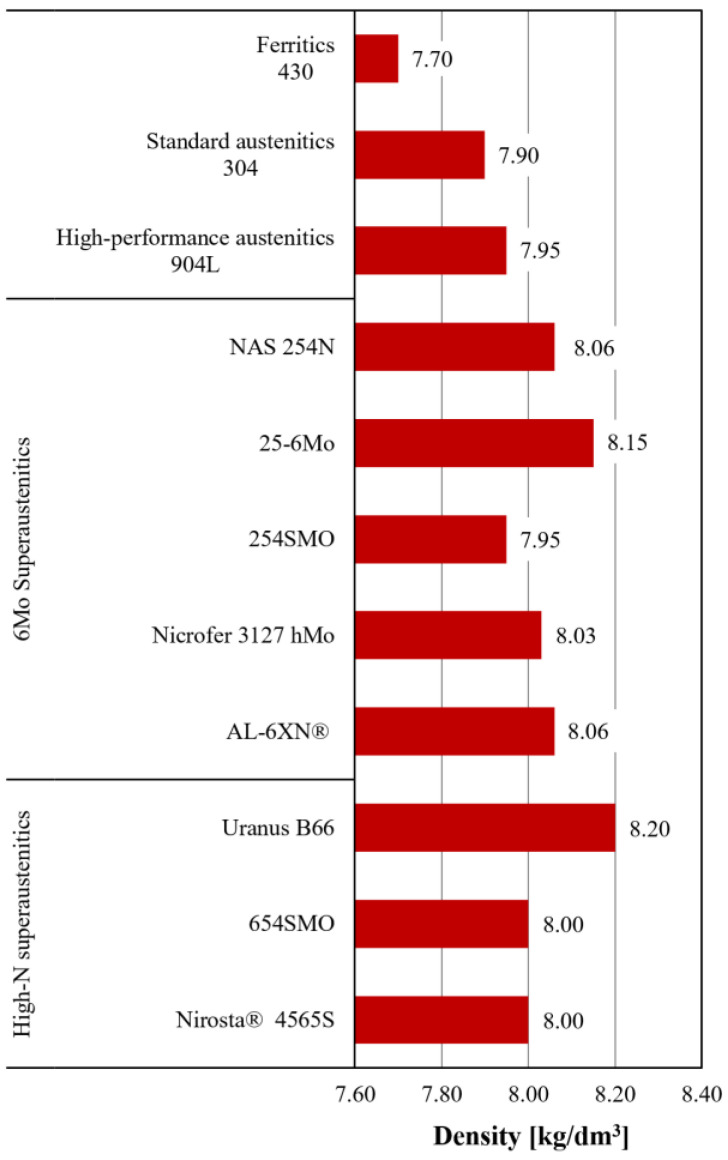
Density values for selected SASSs together with ferritic AISI 430, standard austenitic AISI 304 and high-performance austenitic AISI 904L [3].

Knowledge of physical properties is fundamental in the engineering context, especially because of the implications on product design and manufacturing processes. A review of the most relevant physical properties for SASSs will be given below, starting with the simplest one in terms of definition: density. The density values for selected SASSs are shown in Figure 3, and from the analysis of these data it is easy to see that SASSs are characterized by a higher density on average than other austenitics (both standard and high-performance), and definitely higher than ferritics. The main reason for this difference lies in their chemical composition, and more specifically, in the fact that SASSs are rich in elements with high density values. As shown in Table 1, SASSs contain high amounts of both Ni and Mo which have density values of 8.908 kg/dm^3^ and 10.28 kg dm^3^, respectively [3]. However, the simplistic assumption that density can be estimated as a kind of weighted average among alloying elements is not correct. In fact, other parameters are involved in determining the value, such as the affinity between the elements [16]. Nevertheless, in light of the preceding discussion, it is evident that while an increased content of high-density elements correlates with an increase in density, this relationship is not a simple linear dependence [3]. From a product design point of view, higher density results in an increase in part weight for the same geometry, hence, volume. However, as will be reported in Section 3.2, SASSs are characterized by higher strength values than standard austenitics. This can enable a reduction in thickness while maintaining the same strength, thus allowing a decrease in weight despite the higher density.

While among the physical properties, density plays a major role, especially in design, other physical properties influence the processability of the material. SASSs, like the other stainless steel types, are mostly produced as wrought products and thus need to be hot worked and subsequently heat-treated [3]. Consequently, heating them to the optimum process temperature is required. In this context, two properties show particular importance: thermal conductivity and specific heat. Both of them help in defining the material’s behavior towards high-temperature heating by defining, in the first case, the ease by which heat can flow through it and, in the second, the energy required from a unit quantity of its mass to increase its temperature by one degree. As can be seen from the values in Table 2, SASSs show lower thermal conductivity on average than standard austenitics and, certainly, than ferritics.

Regarding specific heat, excluding the three cases of 254SMO, 654SMO and Nirosta^®^4565S, the values are lower than those of standard austenitics. The differences within the same family of alloys can be traced back to their different chemical compositions. The mean coefficient of thermal expansion (CTE) can be added to the previous two properties in determining important material characteristics related to temperature. In fact, a high value of this parameter influences the dimensional stability of parts under service conditions involving wide temperature variations over time. Additionally, the value of CTE helps in predicting the behavior of welded joints in terms of distortion and possible development of internal stresses.

### 3.2. Mechanical Properties

Mechanical properties are defined by the material’s response to the application of external stresses. Among the most commonly used mechanical properties for the characterization of materials are tensile properties, the values of which for SASSs are given in Table 3.

**Figure 4:**
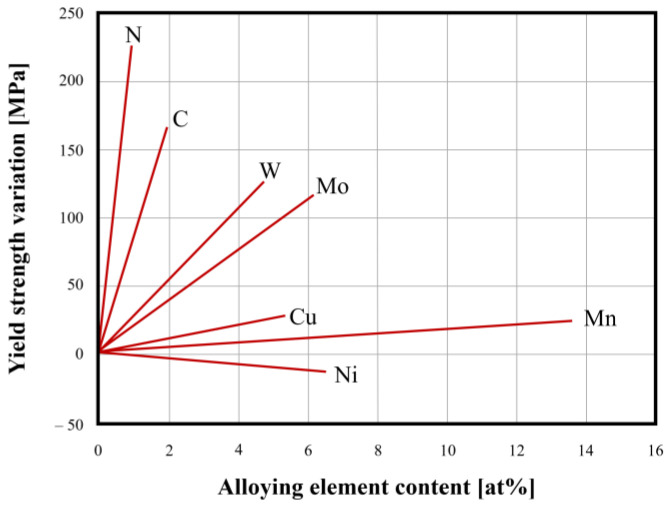
Solid solution hardening effect of common alloying elements in SASSs [3].

From the data given in Table 3, it is easy to see the high-performing behavior of SASSs, especially compared to standard austenitics in relation to a widely used parameter in engineering design: yield strength. SASSs show consistently higher yield strength than standard austenitics, especially in the case of high-N superaustenitics where the value is practically double that of AISI 304. The superior tensile strength characteristics are easily explained by the chemical composition of these steels. In fact, as shown in Table 1, they are rich in solid solution hardening elements that exhibit different strengthening effects as shown in Figure 4. As can be seen in Figure 4, an increase in the percentage in one of the key elements of SASSs, Ni, results in a reduction in yield strength. However, the high content of other strengthening elements counterbalances this tendency and allows an increase in properties. This phenomenon can be attributed particularly to Mo and N. The latter, among the alloying elements of SASSs, is the most strongly associated with the highest yield strength increase per at% added.

Like all austenitic stainless steels, SASSs show a high strain hardening tendency. The strain hardening behavior of austenitic grades increases as the content of specific alloying element increases, in particular when N content is high. It is therefore not surprising how these steels, which can show a N content as high as 0.6%, are characterized by a pronounced strain hardening tendency. This characteristic brings advantages with regard to the strengthening as a consequence of cold working operations but is on the contrary a disadvantage with regard to machinability (as will be explained in the dedicated section) [3]. A last detail related to mechanical properties concerns the mechanical behavior at low temperature. SASSs do not exhibit ductile–brittle transition temperature (DBTT), and therefore, their resistance to dynamic loads (e.g., those employed in the Charpy test) does not drop dramatically with decreasing temperature. Hence, the change in the toughness of the material occurs gradually as temperature decreases.

### 3.3. Corrosion Resistance

Corrosion resistance has been the driving force behind the development of SASSs, with obvious focus on wet corrosion. These materials have a chemical composition designed to surpass the performance of standard austenitics and to bridge the gap with Ni alloys, aiming to be a more cost-effective alternative to the latter and to Ti alloys [3].

Localized corrosion, such as pitting and crevice corrosion are among the most insidious and typical forms of corrosion for alloys with passive behavior such as stainless steels. The prediction of the behavior of stainless steels, and thus SASSs, in terms of wet corrosion resistance in various environments has led to the development of a variety of experimental techniques, as well as indicators useful for ranking the different grades. Without considering electrochemical-related parameters such as the pitting potential, probably the most widely used indicator to express an estimate of the performance of stainless steels is the pitting resistance equivalent (PRE). The PRE is calculated by an empirical formula based on the chemical composition and the contribution of alloying elements to the increase in localized corrosion resistance, i.e., those that have beneficial effects in this regard. The original formula was developed for alloys without N as an intentionally added alloying element and thus omitted it. The PRE equation is given by [3]

(1) $$PRE=\%Cr+\left(3.3\times \%Mo\right)$$

Equation (1) considers only the contribution of Cr and Mo using their weight percentages to obtain the result. Research related to the development and improvement of stainless steels during the 20th century has shown that N also greatly improves localized corrosion resistance. The beneficial effect of N on the corrosion resistance of SASSs has been further underscored by recent investigations. Potentiodynamic tests conducted in 3.5% NaCl solutions have demonstrated that an increased N concentration in the alloy promotes passivity. Specifically, these electrochemical analyses revealed a shift towards lower current densities in the passive region with increasing N content, signifying enhanced passive film stability and, thus, superior resistance to localized corrosion phenomena [17].

In light of the aspects just discussed, it is imperative to also incorporate the contribution of N in the calculation. Therefore, the PRE equation needs to be modified to include N [3]:

(2) $$PREn=\%Cr+\left(3.3\times \%Mo\right)+\left(16\times \%N\right)$$

The pitting resistance equivalent calculated with Equation (2) is named PREn and uses 16 as the multiplication factor for N. Some authors, however, suggest using 30 instead of 16, as is seen in the equation used for another family of alloys: Ni-based alloys. This assumption is justified by the chemical compositional similarities between some Ni-based alloys and SASSs. However, this issue remains a subject of ongoing debate [5].

**Figure 5:**
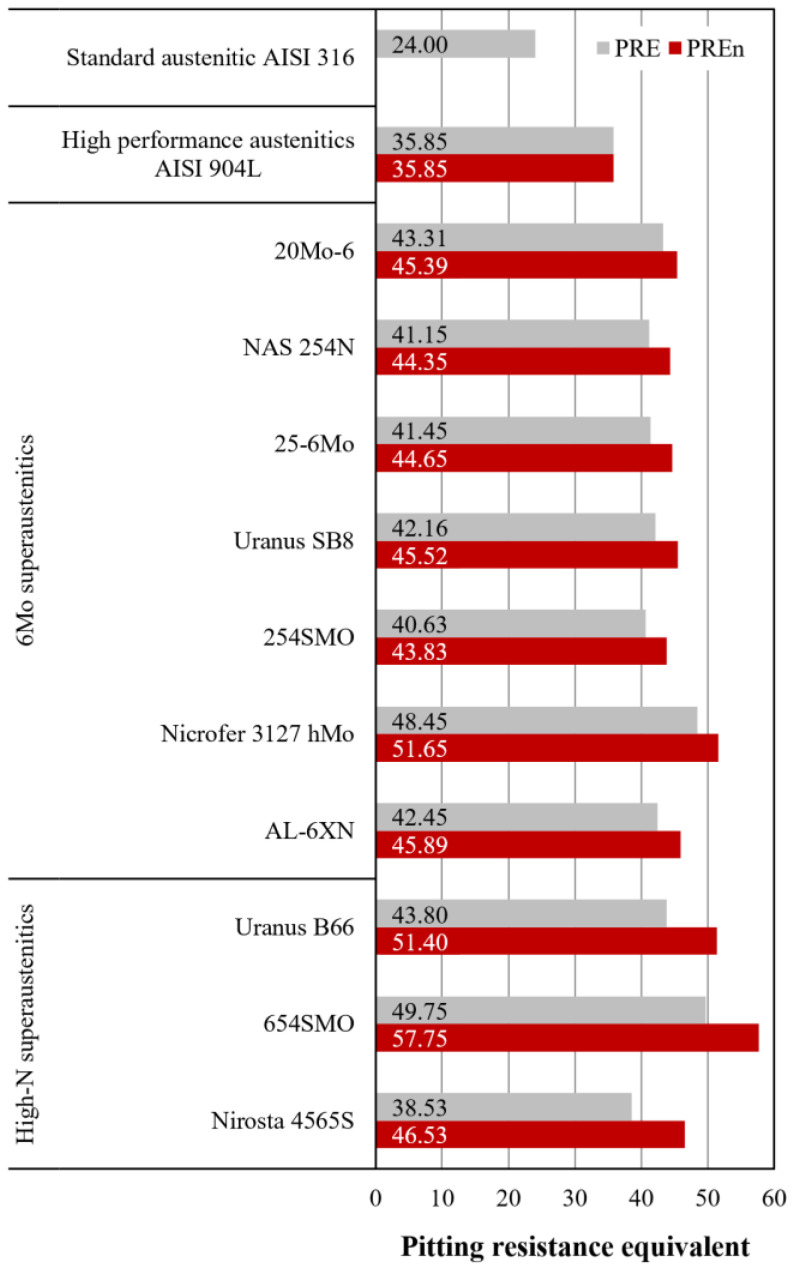
Pitting resistance equivalent calculated for selected SASSs according to Equations (1) and (2). Standard austenitic AISI 316 and high-performance austenitic AISI 904L are given for comparison.

Values of the pitting resistance equivalent calculated according to Equations (1) and (2) for selected SASSs, using the average content of each of each alloying element from Table 1, are provided in Figure 5. From the data given in Figure 5, it is possible to see that the pitting resistance equivalent of SASSs is always considerably higher than that of a standard austenitic such as AISI 316, which still contains Mo, indicating that these steels were also developed to improve resistance to localized corrosion phenomena.

In addition to pitting resistance equivalent, other indicators are also used for estimating resistance against localized corrosion phenomena. Two of the most commonly used values are the critical pitting temperature (CPT) and critical crevice temperature (CCT). In both cases, the parameter expresses the minimum temperature above which the respective localized corrosive phenomenon can occur stably. CPT and CCT are more practically-oriented indicators than pitting resistance equivalent. This is also evidenced by the fact that they are often related to the corrosion rate obtained from tests conducted in accordance with standard procedures such as ASTM G48. In this context, for example, verification of the crevice corrosion rate in seawater according to ASTM G48 shows that alloys with CCT > 35 °C are virtually immune to crevice corrosion. For instance, AISI 904L has a CCT of 15 °C, while 254SMO reaches 38 °C and 654SMO reaches 75 °C. The same comparison made for CPT shows that AISI 904L has a value of 40 °C, 254SMO of 73 °C and 654SMO of 105 °C [3].

Beyond the factors stated for critical pitting temperature and those specified in reference standards, it is crucial to note that CPT is also influenced by the composition of the test solution. For instance, studies determining this parameter on 254 SMO, a common 6Mo superaustenitic stainless steel, revealed a decrease in CPT with increasing NaCl concentration in the test solution. As an example, a 36% reduction in CPT was observed when the NaCl concentration increased from 4% to 30% [18].

**Figure 6:**
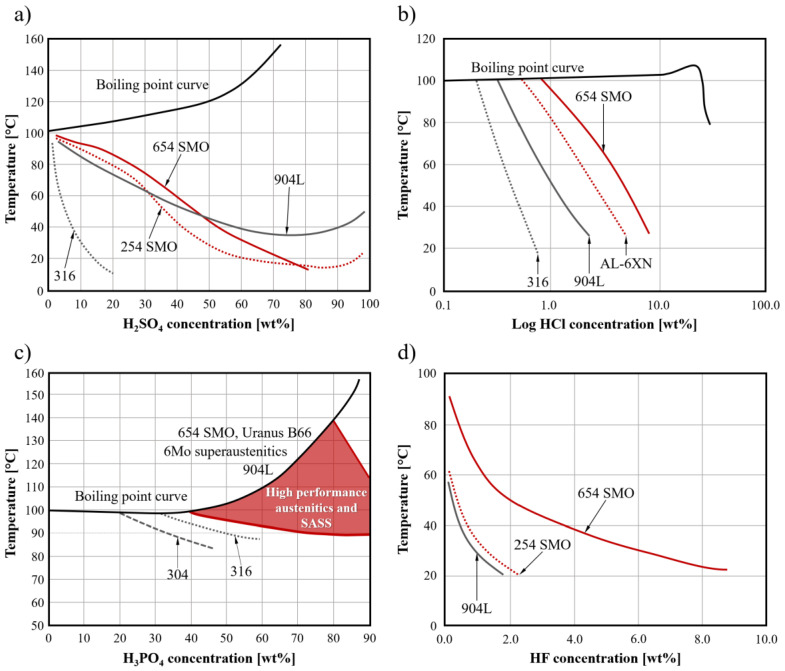
(**a**) 0.1 mm/year isocorrosion curves of various types of stainless steels including SASSs in (**a**) non-aerated H_2_SO_4_ solutions; (**b**) pure HCl acid solutions; (**c**) pure H_3_PO_4_ acid solutions; (**d**) pure HF acid solutions [3].

In addition to the parameters just mentioned, corrosion resistance can also be assessed by evaluating isocorrosion curves when the material is in contact with a specific environment. In this case, the evaluation of corrosion resistance identifies the best-performing materials as those that can be used under the most aggressive conditions while maintaining a corrosion rate below a chosen threshold, typically expressed in mm per year. In this context, some of the most corrosive substances found in a variety of industrial applications are inorganic acids. Among these, H_2_SO_4_, HCl, H_3_PO_3_ and HF are known to be particularly aggressive toward materials [3]. Figure 6a shows the comparison between two members of the SASSs group, AISI 904L and a standard austenitic. It can be seen that for H_2_SO_4_ concentrations above about 48%, AISI 904L performs better than SASSs mainly due to its higher Cu and Ni content compared to 254SMO and 654SMO. At lower concentrations, SASSs show better resistance, especially at higher acid solution temperatures.

In case of contact with HCl-based solutions (Figure 6b), both members of the 6Mo group and the more highly alloyed high-N 654SMO perform better than AISI 904L and, of course, AISI 316.

Even in the case of H_3_PO_4_ solutions (Figure 6c), SASSs demonstrate the ability to withstand significantly higher concentrations and temperatures than standard austenitics.

Finally, regarding resistance in HF solutions (Figure 6d), SASSs perform only marginally better than AISI 904L among members of the 6Mo group. The situation is markedly different for high-N SASSs, where it can be seen that 654SMO can tolerate more than three times the concentration at significantly higher temperatures.

The high corrosion resistance of SASSs is maintained even in solutions with compositions that include more substances potentially hazardous to standard austenitics. This is evidenced, for example, by recent studies using potentiodynamic polarization techniques on AL-6XN^®^ PLUS in a solution of 0.5 mol/L H_2_SO_4_ + 0.5 mol/L NaCl. AL-6XN^®^ PLUS is a version of AL-6XN^®^ with Cr, Mo and N contents at the top of the range given in Table 1. Comparison with AISI 304L, AISI 316L and AISI 317L showed that the passive region for AL-6XN^®^ PLUS is significantly more stable than that observed for the other austenitic steels tested. Furthermore, AL-6XN^®^ PLUS was the only steel in the tested group that did not exhibit localized corrosion, while the others were all susceptible to pitting corrosion [19]. In addition, SASSs demonstrate superior corrosion resistance even in solutions combining multiple acids. The research involving exposure to modified green death solutions, designed to simulate the environment in desulfurization devices of ship exhaust systems (scrubbers), has shown significantly better behavior of SASSs compared to standard austenitics. Specifically, the modified green death solution consists of H_2_SO_4_ (16.9 vol%), HCl (0.35 vol%) and H_2_O. It was found that under the most severe test conditions, AL-6XN^®^ exhibited a 4.5 times lower corrosion current than AISI 316L for the same exposure to the corrosive environment [20].

In addition to the exposure to corrosive environments, the evaluation of corrosion resistance must also consider forms that combine corrosive environment with applied external stresses, typically of the tensile type, i.e., mechanochemical corrosion. One of the most insidious forms of mechanochemical corrosion is stress corrosion cracking (SCC). The performance of SASSs against SCC is superior to that of standard austenitics, primarily due to their higher Cr, Ni and Mo content. A common method for effectively representing a material’s behavior against SCC is to refer to rankings obtained through tests that simulate the combined effects of a corrosive environment and applied stress. One such possibility is the drop evaporation test (DET) [21], conducted according to the ISO 15324:2000 standard. This test also enables the determination of a load value above which crack initiation occurs [22].

**Figure 7:**
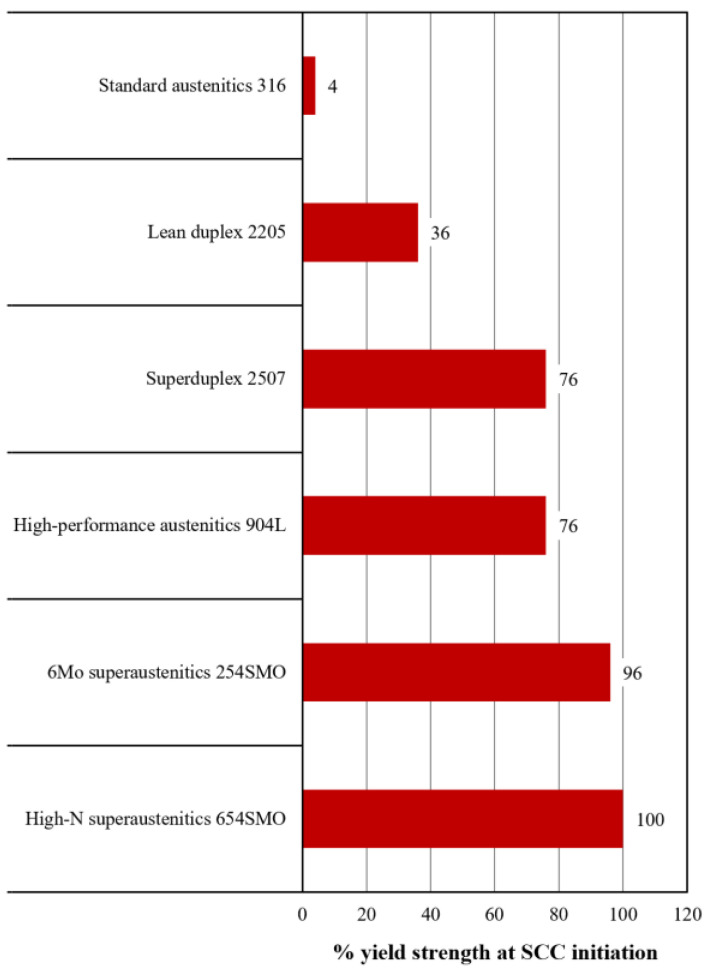
Percentage of the yield strength at which crack initiation takes place during DET test as indication of the SCC resistance of various stainless steels [3].

In this context, the values obtained from DET for SASSs in comparison with other stainless steels are shown in Figure 7. In order to provide a broader comparison, the lean duplex 2205 and superduplex 2507 grades are also included in Figure 7. These materials, particularly 2507, have an excellent combination of mechanical strength and corrosion resistance. In addition, they exhibit higher resistance to SCC than standard austenitics due to their chemical composition and dual-phase microstructure [5]. As can be seen from Figure 7, SASSs perform better even than duplex, achieving crack initiation stress levels under the same corrosion conditions that approach their yield strength. This result, when compared with the values associated with AISI 316, once again highlights how these steels can be a valuable choice for extreme operating conditions in terms of corrosive environment and strength requirements.

## 4. Manufacturing, Processing and Applications

**Figure 8:**
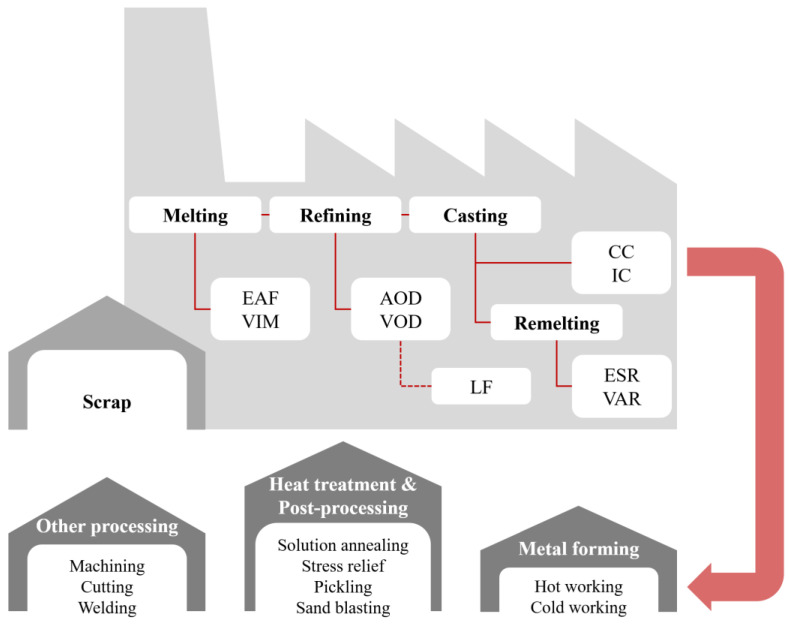
Typical processing route in the manufacturing of products made of SASSs obtained from wrought products having scrap as starting raw material [3].

Each material has peculiar properties and characteristics that help define the technologies and processes best suited to constitute the pathway for obtaining products. The choice of the manufacturing pathway is a nontrivial combination that depends on the physical, mechanical and technological properties of a material; the production volumes; the quality required in terms of acceptable scrap rate; and finally, the right balance of resulting costs. In this context, Figure 8 shows the typical processing route for products made from SASSs. The operational flow described refers to the usual pathway for products made from wrought products. This is because cast products constitute only a small portion of the SASSs landscape and, for the purpose of providing a comprehensive overview, are of limited significance [3].

### 4.1. Melting, Refining and Casting

As shown in Figure 8, the starting point for the production of SASSs is the melting of scrap in an electric arc furnace (EAF) [3]. An EAF is considered one of the most important facilities in stainless steel production companies and, nowadays, is used by more than 80% of stainless steel producers that use scrap as raw material. The rest is produced using mainly induction furnaces and, in limited cases, other less common practices [23,24]. Concerning SASSs, the EAF charge is composed scrap with composition calibrated according to the downstream refining process. However, scrap composition cannot be the same as that of the final product because of the likely oxidation processes involving Cr and Mo during the melting process. The scrap should contain Cr and Mo, and appropriate calibration of the process temperature, as well as the composition and reactivity of the slag, will be key parameters for limiting oxidation of these elements. An alternative melting process to EAF that is more expensive but can provide more accurate control of chemical composition combined with higher cleanliness would be vacuum induction melting (VIM). In this case, in view of the fact that melting takes place under vacuum, the scrap used has the same composition as the material to be produced. However, due to the intrinsic limitations in chemistry adjustment possibilities during the process and related costs, VIM is rarely employed in the production of SASSs. In some cases, however, it is used as a secondary process to improve cleanliness and the associated properties (e.g., corrosion resistance) [3].

The refining process of SASSs typically employs the AOD converter, which is essential for their development. Its introduction into industrial practice made it possible to produce steels with high contents of the key elements of SASSs: Cr, Mo, N and Ni. For steels with high N content such as SASSs, and in particular the members of the high-N group, it is paramount to successfully retain high atomic N content in the liquid while preventing it from recombining into N_2_ or being lost to the atmosphere through bubbling. To accomplish this, during the AOD process, manufacturers operate continuous N injection in combination with a high Cr content in the liquid as this element is able to retain atomic N in the liquid, thus preventing its recombination or its loss. In addition to N, Cr and Mo must also be maintained at the right concentration in the liquid, and a key step lies in the optimal control of the characteristics of the slag floating on the liquid. Specifically, the reactivity of the slag must be calibrated by properly saturating it so that it will be unable to remove excessive amounts of Cr and Mo from the liquid. Last but not least, temperature control throughout the process is also critical. This is because temperature affects the stability of oxides, the reactivity of the slag and also the behavior of N. For premium-quality materials, which are subject to stringent market-driven compositional requirements, one approach to further enhance the conversion process is to use vacuum conversion, such as vacuum oxygen decarburization converter (VOCD) or vacuum oxygen decarburization (VOD). These processes not only offer higher C removal rates than AOD but are attractive for SASSs because of the possibility of achieving lower values of S concentration. This element is particularly detrimental to SASSs as it reduces their high-T ductility and promotes hot cracking during solidification or welding [3].

With regard to post-refining operations, the casting practices relevant to SASSs are those aimed at producing semi-products intended for subsequent hot working operations. In this context, the processes typically employed are ingot casting (IC) and continuous casting (CC). As previously mentioned, the market of cast products is relatively small and will not be considered further here. Regardless of whether IC or CC is used, the main issue concerning the solidification of SASSs is the occurrence of segregation phenomena and their consequences. If this inhomogeneity is confined to the microstructural dimension, it is referred to as *microsegregation*. Conversely, when segregation extends over large distances, from a few millimeters up to the size of the entire ingot, billet or slab; it is termed *macrosegregation* [3]. As discussed in previous sections, among the alloying elements in SASSs, Cr and Mo exhibit the highest tendency to segregate during solidification, with Mo being the most prone. During solidification, both Cr and Mo atoms tend to segregate in the interdendritic regions (IDRs), promoting the formation of eutectics or secondary phases, all of which are generally detrimental to the material [25,26]. In fact, the main consequence of segregation is a non-uniform elemental distribution, which may locally reach eutectic, eutectoid or simply limited solubility conditions resulting in the precipitation of secondary phases. Referring also to Figure 2, the phases that may form during the solidification of SASSs due to segregation, in addition to austenite (γ), include δ ferrite, σ phase in eutectic form with γ or in the eutectoid form with γ_2_ (also known as eutectoid γ) resulting from the decomposition of δ ferrite. Unfortunately, significant Mo segregation is virtually unavoidable in ingots produced by IC due to the relatively slow cooling rates inherent to the process. Although CC offers higher cooling rates compared to IC, these rates are still insufficient to fully suppress segregation. If macrosegregation occurs, the issue becomes practically irresolvable, as such inhomogeneity cannot be eliminated even through the solution annealing treatments commonly applied to mitigate/eliminate microsegregation [3,26].

Over time, the casting process of SASSs has been optimized to minimize macrosegregation by adjusting process parameters that influence its formation. In addition to carefully calibrating the cooling rate, effective control of liquid steel temperature before casting, casting speed and liquid flow behavior in the mold (e.g., through electromagnetic stirring) are all critical factors [3]. The effects of excessive microsegregation become particularly evident during hot working following casting. The enrichment of solute atoms in IDRs gives rise to deformation-aligned band patterns known as *microstructural bandings* (MSBs). These bands, typically enriched in the brittle σ-phase, compromise mechanical properties, corrosion resistance and the effectiveness of grain refinement during recrystallization [26]. As previously mentioned, the tendency of an element to segregate is strongly influenced by the cooling rate during solidification. An absence of segregation corresponds to a partition coefficient of 1. Numerous studies on SASSs have demonstrated that increasing the cooling rate reduces segregation, as limited atomic diffusion brings the partition coefficient closer to unity. Various strategies can be adopted to increase the cooling rate, depending on whether the context is experimental or industrial. In industrial-scale flat product production, one possible method is *twin-roll strip casting* (TRSC). TRSC involves the direct solidification of liquid steel between two counter-rotating, water-cooled rolls, forming a mold. This process enables high cooling rates and applies compressive stress that disrupts the initial solidification structure [3,25,27].

In light of the challenges associated with segregation, an alternative approach for producing SASSs with homogeneous composition and microstructure involves the use of remelting processes. Specifically, the two technologies most commonly employed are *electroslag remelting* (ESR) and *vacuum arc remelting* (VAR). In both cases, the primary material is remelted and allowed to resolidify under controlled cooling conditions. In the ESR process, the molten alloy passes through a reactive slag designed to capture impurities. The slag acts as a filter, removing undesirable elements while allowing the purified melt to resolidify in a mold under a defined cooling regime. In the case of VAR, remelting and resolidification occur under vacuum in a layer-by-layer fashion, with inherently high cooling rates. This configuration helps to eliminate macrosegregation, minimize microsegregation and improve overall material homogeneity. Remelting processes are typically reserved for the production of high-performance components, where the stringent requirements for microstructural cleanliness, chemical uniformity and defect minimization justify the higher production costs. Due to the advanced equipment involved and the superior quality of the resulting products, remelted SASSs command significantly higher market prices compared to those produced via conventional casting methods [3].

### 4.2. Metal Forming and Heat Treating

Austenitic stainless steels are known to have excellent formability characteristics with regard to plastic deformation, both in terms of hot working and cold working operations [23]. Although SASSs belong to this class of steels and share *fcc* crystal structure, their forming behavior differs significantly and is, in many respects, more closely aligned with that of Ni-based alloys. This divergence is primarily attributable to their distinct chemical composition (see Table 1), which includes elevated concentrations of alloying elements that promote solid solution strengthening (Figure 4) [3].

**Figure 9:**
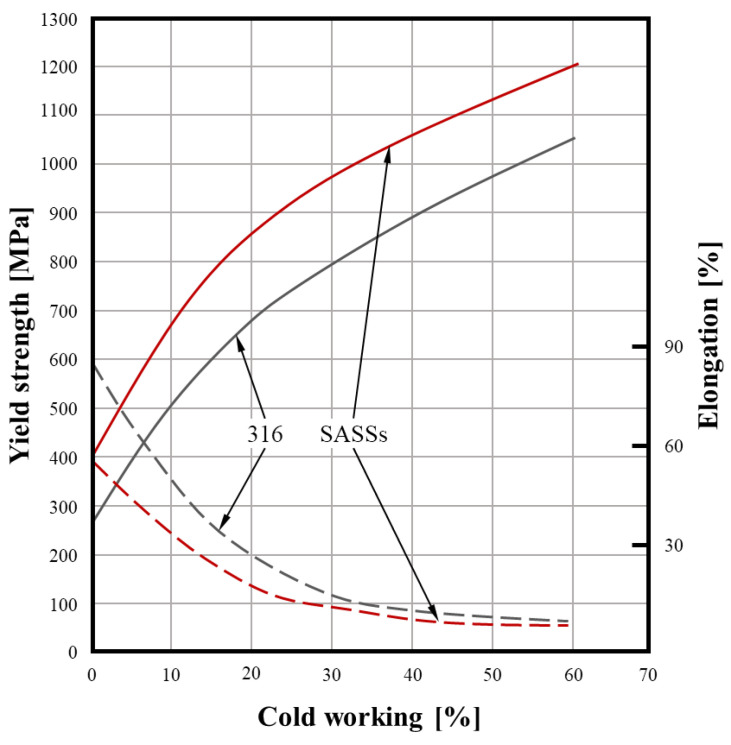
Comparison of the effects of cold working between the standard austenitic AISI 316 and the typical behavior of SASSs in terms of yield strength and elongation at rupture [3].

As a result, SASSs exhibit higher mechanical strength than standard austenitics and also display pronounced work-hardening behavior during cold forming operations [28,29]. These characteristics must be carefully considered when selecting or designing forming processes for SASSs, especially in applications requiring tight dimensional tolerances or complex geometries [3]. This behavior becomes particularly evident when examining the data presented in Figure 9, which compares the typical response of SASSs with that of a standard austenitic stainless steel such as AISI 316. Although both materials exhibit a similar trend in yield strength increase with progressive cold deformation, SASSs consistently display higher absolute values, reflecting their enhanced mechanical strength. Conversely, ductility, expressed as elongation at break, tends to be lower for SASSs at equivalent levels of cold work. This reduction in ductility is directly related to their higher intrinsic mechanical properties (see Table 3) and greater strain hardening capacity These mechanical characteristics have notable implications for processability. While SASSs can be cold formed using conventional techniques, their elevated strength and work-hardening behavior necessitate careful adaptation of processing methods and equipment. For instance, in bending operations, the pronounced springback observed in SASSs requires substantial overbending to achieve the intended geometry. In cold rolling operations, commonly used for the production of flat products, the higher forces and energy input required for plastic deformation typically demand the use of cluster-type rolling mills to ensure effective material processing [3].

Hot working of SASSs involves complex considerations as well. The research on these steels has predominantly concentrated on aspects such as mechanical properties, corrosion resistance and the precipitation of secondary phases, resulting in a relative scarcity of data concerning their hot workability. This knowledge gap is further compounded by the proprietary nature of industrial processing routes, which are often withheld by manufacturers due to their strategic value [3,30]. In general, SASSs exhibit significantly higher strength at elevated temperatures compared to standard austenitic stainless steels. This is primarily attributed to their elevated contents of Cr, Mo and N. As a result, their hot working behavior more closely resembles that of Ni-based alloys. This similarity implies the need for dedicated equipment capable of applying higher forces, as well as withstanding the increased reaction of the material during processing [3,31].

The deformability characteristics of SASSs are strongly influenced by their chemical composition and generally remain inferior to that of standard austenitic stainless steels when dealing with hot working operations. This turns out to be proportional to the performance level of the material considered. For instance, 654SMO, among the highest-performing grades in terms of corrosion resistance, is also among the most challenging to hot work, exhibiting limited formability and a pronounced tendency toward hot cracking, particularly edge cracking. To ensure successful hot working operations on these materials and to obtain defect-free product, it is essential to understand the specific hot working behavior of each alloy. In this regard, the development of processing maps turns out to be a practically inevitable step for optimal calibration of the process temperature and strain rate [30].

**Figure 10:**
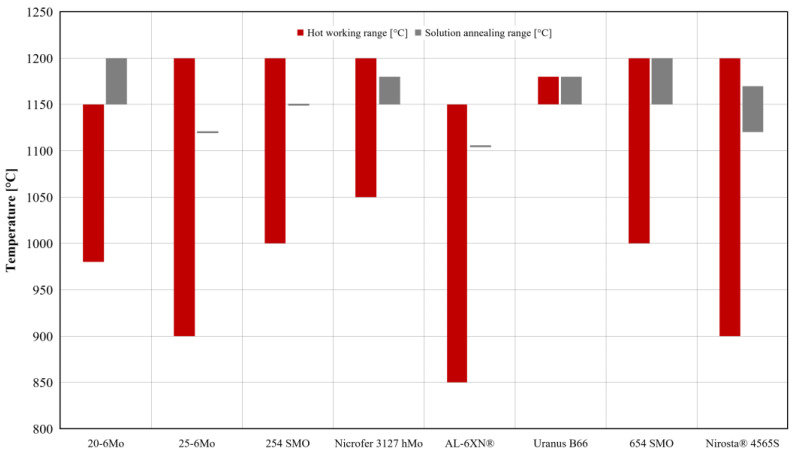
Typical temperature range for hot working and solution annealing heat treatment of selected SASSs [3].

The challenges associated with hot working of SASSs are not limited to the deformation stage but also include critical issues arising during the reheating phase required to reach the appropriate processing temperature. As illustrated in Figure 10, the hot working temperature windows for SASSs vary by alloy grade but typically exceed 1100 °C [3]. SASSs were not specifically engineered for high-temperature corrosion resistance; thus, exposure to such environments promotes surface degradation primarily due to high-temperature oxidation phenomena. Notably, the formation of volatile compounds such as CrO_3_ and MoO_3_, originating from the oxidation of chromium and molybdenum, two key alloying elements in SASSs, leads to surface deterioration. This oxidation-induced loss not only alters the surface morphology but may also compromise the integrity and performance of the material during subsequent processing steps [32]. Under limited exposure, or with reheating in controlled atmosphere furnaces, the formation of these compounds does not lead to particular problems. Conversely, reheating in oxidizing atmosphere and for excessively long exposure can lead to markedly enhanced formation of volatile CrO_3_ and MoO_3_. This phenomenon results in a chemically altered surface layer, depleted in alloying elements, that differs in both composition and mechanical behavior from the bulk material. This can lead to the occurrence of defects such as surface decohesion during plastic deformation processes (e.g., hot rolling or forging). To mitigate such issues, the exposure of SASSs to high temperatures should be minimized and limited to the time strictly necessary to achieve thermal homogeneity throughout the material. The optimization of the reheating cycle and schedule must consider not only the risks of high-temperature oxidation but also the thermal properties of the alloy (see Table 2). This is particularly critical for grades with low thermal conductivity and high specific heat capacity, such as 654SMO [3].

This issue is also reflected in a fundamental process that occurs after both cold and hot working: heat treatment. SASSs can be subjected to two types of heat treatments: stress relief annealing and solution annealing. Stress relief annealing is usually applied following cold working and involves heating the material to relatively low temperatures, typically not exceeding 450 °C, depending on the residual stress level to be relieved. Short-duration treatments at higher temperatures are also possible, though these may introduce surface oxidation issues. Solution annealing, on the other hand, involves significantly higher temperatures, comparable to those used in hot working operations (see Figure 10). The primary objectives of this treatment are to homogenize the microstructure through a combination of recrystallization and dissolution of the secondary phases (see Figure 2) that have formed as a consequence of non-equilibrium cooling after hot deformation. The duration of the heat treatment must be carefully optimized to achieve full dissolution of undesirable phases while avoiding excessive surface oxidation and limiting grain growth.

Heat treatment is always followed by rapid cooling, usually in water. This step is necessary for preventing free cooling from being too slow and having a cooling trajectory that crosses the precipitation lines of secondary phases in the relevant TTT or CCT diagram [3,11,13]. However, in some cases, the solution annealing treatment is not fully effective in dissolving the secondary phases formed during prior hot working processes. This is partly attributed to the inherent limitations of the treatment in achieving complete dissolution of all pre-existing secondary phases, and partly to potential non-uniformities during the cooling stage, which can locally create conditions that promote precipitation. Consequently, the presence of isolated colonies of intermetallic SASSs is generally tolerated up to a threshold value defined by the relevant technical specification for the specific product.

### 4.3. Welding

**Figure 11:**
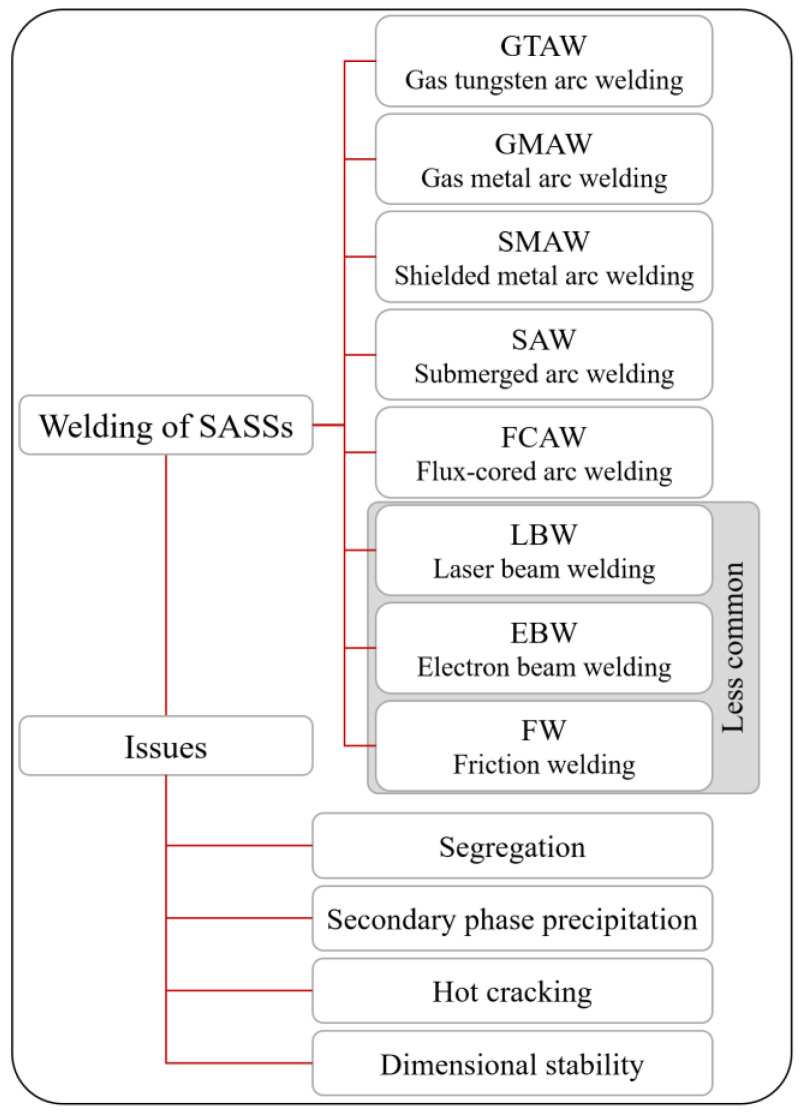
Welding techniques for SASSs and most important related issues [3,33,34].

SASSs can be welded using most conventional welding techniques, as illustrated in Figure 11 [3,32,33]. However, their complex chemical composition introduces several critical challenges. For instance, autogenous welding is generally not recommended for SASSs. Nonetheless, successful autogenous welds have been achieved in thin sections (thickness < 2 mm), provided that an appropriate shielding gas is employed. In such cases, despite the limited volume affected by heating, melting and resolidification, a post-weld solution annealing treatment is necessary to reduce microsegregation and restore a homogeneous microstructure in the weld zone. This step is essential to recover corrosion resistance levels comparable to those of the base material [33]. An exception to these constraints is represented by high energy density welding techniques, such as laser beam welding (LBW) and electron beam welding (EBW). For instance, EBW performed without filler material has demonstrated high weld strength and limited issues, largely due to its low heat input and regularity of the weld bead associated with the technique [4].

A process closely related to autogenous welding, though categorized as solid-state welding, is friction welding (FW). Although not commonly used for SASSs, several studies have shown its feasibility. Specifically, defect-free, with no N-depletion issues welds have been reported for 654SMO, and successful application of the technique to 254SMO has also been documented. In both cases, the resulting weld microstructure was characterized by fine grains and partial recrystallization [35].

All other welding techniques shown in Figure 11 require the use of a filler metal. In the case of SASSs, this necessitates the selection of a filler material that is compatible with their solidification behavior, particularly with regard to the segregation tendency of certain alloying elements. As mentioned in Section 2, among the alloying elements in SASSs, Mo shows the highest tendency to segregate in the liquid during solidification. To avoid Mo-depleted weld joints with jeopardized corrosion resistance, a commonly accepted guideline is to use a filler metal containing at least 1.5 times the Mo content of the base metal. Consequently, filler materials typically adopted for welding SASSs are not Fe-based alloys, but rather Ni-based alloys, such as Alloy 625, Alloy 686, or Alloy C22 [27]. In fact, compared with steels, Ni-based alloys have indeed a higher solid solubility of Mo reaching maximum possible values of 25% [34]. Additionally, they contain high levels of Cr and use Ni as the base element, making them well-suited as fillers for joining alloys with similarly high Cr and Ni content, such as SASSs.

**Figure 12:**
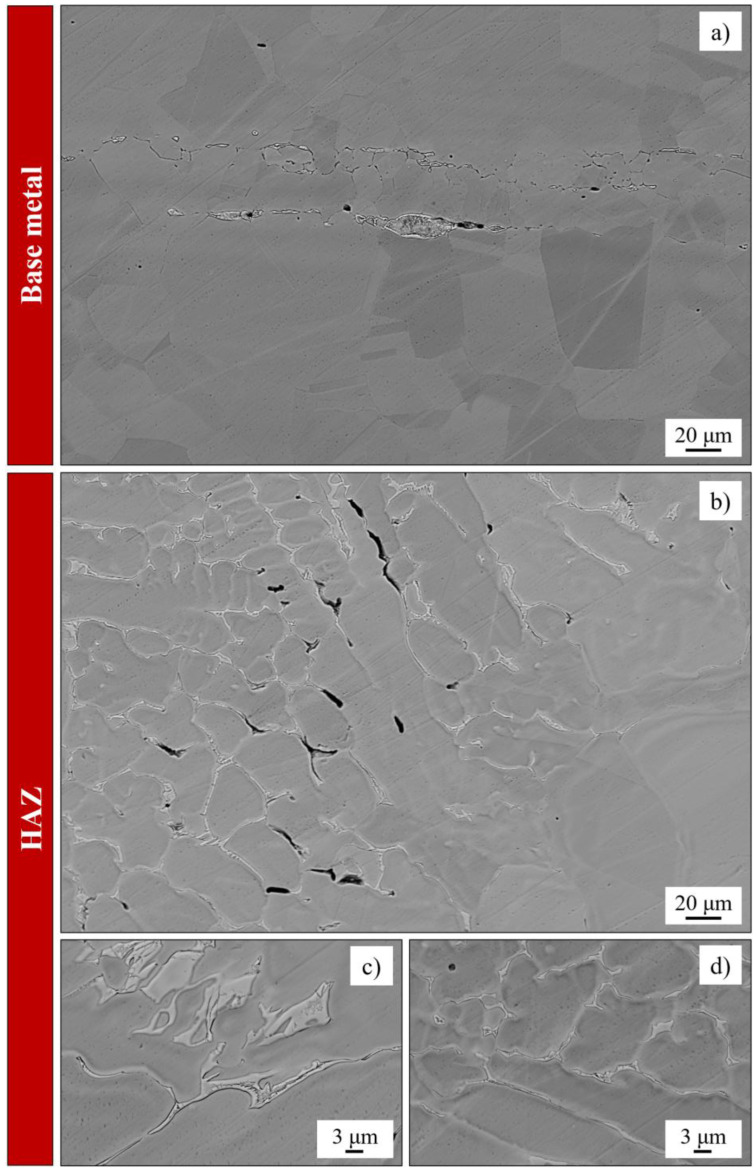
Backscattered electrons SEM micrography of a 254SMO sheet subjected to GTAW without filler material. The weld consists of a single spot where the torch remained stationary for 30 s to promote the precipitation of secondary phases; (**a**) base metal in the solution annealed condition showing the typical grain morphology of the *fcc* matrix of SASSs together with isolated intermetallics that were not dissolved by the heat treatment; (**b**) HAZ near the fusion zone exhibiting heavy precipitation of intermetallic compounds; (**c**) detailed view of the intermetallics observed in the HAZ; (**d**) further detailed view of intermetallics observed in the HAZ. Micrographs obtained using a Zeiss EVO MA15 LaB6 microscope (Oberkochen, Germany) equipped with QBSD detector [images courtesy of Omeco Srl].

Unfortunately, the formation of Mo-depleted zones is not the only segregation-related concern. Although to a lesser extent than Mo, Cr also tends to segregate during solidification, thereby promoting the development of Cr-depleted regions and formation of Cr-containing phases such as carbides. As shown in Figure 2, the chemical composition of SASSs inherently predisposes them to the precipitation of secondary phases during welding such as σ and χ. Furthermore, as explained in Section 4.2, intermetallics that were not dissolved by the heat treatment may persist even in the solution-annealed material prior to welding, as shown in Figure 12a. As illustrated by Figure 12b–d, these phases primarily form in the heat-affected zone (HAZ), where the thermal exposure is sufficient to trigger their nucleation and growth. In contrast, the weld seam itself is generally less susceptible to phase precipitation due to the higher peak temperatures and rapid cooling rates that suppress such transformations. However, this assumption becomes invalid in multi-pass welding operations. The relatively low thermal conductivity of SASSs (see Table 3) inhibits efficient heat dissipation, causing prolonged thermal exposure in the HAZ. This extended exposure promotes the precipitation of secondary phases such as those seen in Figure 12 [2,36]. The precipitation is typically heterogeneous across the HAZ, with different phases forming depending on the local thermal gradient and distance from the fusion line. The literature indicates that the formation of the σ phase is nearly unavoidable in the HAZ. Furthermore, in alloys such as 654SMO, the R phase has also been observed in regions farther from the weld bead [36]. In addition to this, despite the low C content of SASSs, carbides can also form during cooling. The most common type is M_23_C_6_, but there is evidence that SASSs can also form M_6_C [3]. The precipitation of secondary phases during welding invariably leads to a reduction in corrosion resistance and a decrease in ductility of the material. These effects are detrimental as they compromise the structural integrity of the welded joint and significantly increase the risk of in-service failure [3,36,37].

As illustrated in Figure 11, an additional critical issue in the welding of SASSs is their susceptibility to hot cracking. This phenomenon is primarily linked to the presence of residual levels of P and S, as well as the absence of δ ferrite, as discussed in Section 2. Despite the extremely low impurity content typically achieved in SASSs, hot cracking remains a major concern, particularly because the filler materials employed do not promote δ ferrite formation. In these steels, hot cracking may manifest during solidification (solidification cracking) or during subsequent welding passes (reheat cracking) [3,38].

Finally, special consideration must be given to post-weld geometry-related issues. As shown in Table 2, SASSs are characterized by a higher coefficient of thermal expansion compared to ferritic stainless steels. This property significantly affects their behavior during cooling and contributes to an increased tendency toward distortion following welding. In contrast, the thermal conductivity of SASSs (also reported in Table 2) is not only substantially lower than that of ferritic grades but also lower than that of standard austenitic stainless steels. As previously discussed, this limits the material’s possibility to dissipate heat effectively. As a result, the heat input during welding tends to remain concentrated near the weld zone, creating steep thermal gradients. This localized heating, combined with the elevated thermal expansion coefficient, amplifies shrinkage stresses and consequently heightens the risk of post-weld distortion [3].

Despite the various challenges associated with welding SASSs, these materials can nonetheless be successfully joined using appropriate techniques. As with all high-alloy stainless steels, the welding of SASSs requires careful control of process parameters and strict adherence to best practices specific to stainless steel welding. Key factors for achieving high-quality welds include the design of suitable joint geometries, the control of interpass temperatures and the use of low heat input, preferably through high energy density welding methods. These measures help mitigate the risks of segregation, phase precipitation and distortion, ultimately ensuring satisfactory performance of the welded joints [26].

### 4.4. Market Share and Applications

The demand for superaustenitic stainless steels (SASSs) is predominantly driven by specific project requirements, despite the presence of stockholders and manufacturers specializing in these alloys. These entities encounter ongoing challenges in determining the optimal type and quantity of products to maintain in inventory. Due to this project-specific nature of the demand, achieving consistent and predictable procurement patterns for specific SASS grades is challenging, although certain grades exhibit higher and more frequent demand than others [3].

Based on recent market evaluations, SASSs are estimated to constitute no more than 0.3% of the global stainless steel production. Considering the average stainless steel world production in recent years, this results in an annual production of around 160,000 t [3].

**Figure 13:**
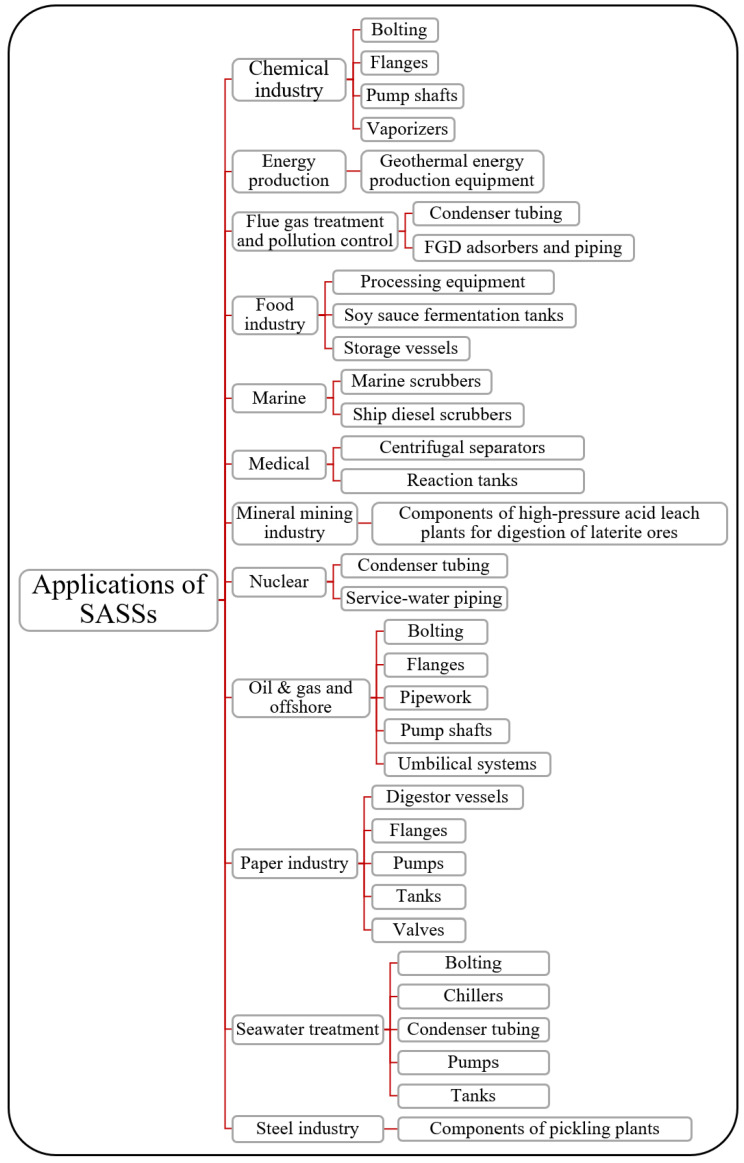
Application sectors and products that can be manufactured with SASSs [3,41].

In terms of applications, SASSs often result in competition with Ni-based alloys, Ti alloys and even superduplex or hyperduplex stainless steels. This is because of their mechanical strength and corrosion resistance characteristics and because of the extreme applications where they can be used [3]. The industrial sectors where SASSs find applications are among the most demanding in terms of performance and are summarized within Figure 13. Application possibilities range from the chemical and energy industries to the nuclear and steelmaking sectors. Unlike other materials with which SASSs could compete for specific applications, such as Ni alloys or Ti alloys, there is as yet no evidence of the actual possibility of using additive manufacturing technologies to make products from SASSs at the present date, except what has been proposed by the company EOS GmbH [3,39]. The company offers the possibility to produce components from 254SMO, using powder compliant with the EN 10088-3 standard. This powder exhibits a particle size distribution between 20 and 65 μm and a declared porosity ranging from 0.01% to 0.02%. The components are fabricated using direct metal laser sintering (DMLS) technology [39]. DMLS operates on the principle of direct laser fusion of metal powder, classifying it within the powder bed fusion (PBF) category according to additive manufacturing standards. Its development began in 1994; therefore, this technology constitutes the first commercial single-process additive manufacturing method for producing metal components such as tools, molds, medical implants and aerospace products [40]. It is therefore plausible that the application of SASSs in additive manufacturing is currently in its nascent phase. This will for sure necessitate further development, particularly to broaden the applicability of this technology to other members within this material family.

## 5. Conclusions

Superaustenitic stainless steels (SASSs) represent a compelling class of materials for demanding engineering applications due to their exceptional balance of mechanical strength, corrosion resistance and cost-effectiveness when compared to other materials with similar properties such as Ni and Ti alloys. While Ni-based alloys offer superior performance in extremely corrosive and high-temperature environments, and Ti alloys offer excellent strength-to-weight ratios and specific corrosion resistance, SASSs can provide a cost-competitive alternative without significant compromise in many critical service conditions, excluding high-temperature applications. Specifically, SASSs exhibit enhanced resistance to localized corrosion, such as pitting and crevice corrosion, in chloride-containing media compared to standard austenitic stainless steels, often approaching the performance of certain Ni alloys. Furthermore, their higher strength compared to conventional austenitics can allow for thinner sections and reduced weight in structural applications, potentially bridging the gap with Ti alloys in specific designs where extreme weight savings are not the primary requirement. This combination of enhanced performance over standard stainless steels and a more favorable cost profile compared to high-performance Ni and Ti alloys places SASSs as an economically attractive and technically viable material choice for a wide range of industrial sectors.

The resistance of SASSs to localized corrosion in seawater and chloride-containing media will continue to make them crucial materials for offshore platforms, seawater treatment systems and other critical applications. Finally, with the transition towards cleaner energy sources, SASSs may find new applications in emerging technologies such as geothermal power plants, energy storage systems and biorefineries, where resistance to corrosion under challenging conditions is paramount.

## Figures and Tables

**Table 2:** Thermal conductivity, specific heat and mean CTE of selected SASSs together with data related to ferritic AISI 430, standard austenitic AISI 304 and high-performance austenitic AISI 904L.

Group	Most Common Name	Thermal Conductivity at 20 °C [W/(m · K)]	Specific Heat [J/(kg · K)]	Mean CTE at T = 20–200 °C [10^−6^ · K^−1^]	Mean CTE at T = 20–400 °C [10^−6^ · K^−1^]
Ferritics	AISI 430	25	460	10.0	10.5
Standard Austenitics	AISI 304	15	500	16.5	17.5
High-performance austenitics	AISI 904L	12	461	16.1	16.9
6Mo superaustenitics	NAS 254N *	11.9	457	15.2	15.8
	25-6Mo	12	461	16.1	16.9
	254 SMO	14	498	17.0	18.0
	Nicrofer 3127 hMo	12	440	14.7	15.5
	AL-6XN^®^ **	13.7	461	15.3	16.0
High-N superaustenitics	Uranus B66 ***	12	450	15.0	16.0
	654 SMO	8.6	510	15.4	16.2
	Nirosta^®^ 4565S	14.5	510	15.5	16.8

* Mean CTE related to T = 30–200 °C and 30–400 °C. ** mean CTE related to T = 20–100 °C and 20–500 °C. *** mean CTE related to T = 20–100 °C and 20–300 °C [3].

**Table 3:** Mechanical properties of selected SASSs at room temperature together with data related to ferritic AISI 430, standard austenitic AISI 304 and high-performance austenitic AISI 904L. Data for all austenitic grades refer to the solution annealed condition [3].

Group	Most Common Name	UTS [MPa]	Yield Strength [MPa]	Elongation [%]
Ferritics	AISI 430	430	260	18
Standard austenitics	AISI 304	520	200	45
High-performance austenitics	AISI 904L	530	230	35
6Mo superaustenitics	25-6Mo	650	295	35
	254SMO	650	300	35
	Uranus SB8	550	250	35
	Nicrofer 3127 hMo	650	280	35
	AL-6XN	690	310	30
High-N superaustenitics	Uranus B66	750	420	35
	654SMO	750	430	35
	Nirosta 4565S	800	420	35

## Data Availability

No new data were created or analyzed in this study. Data sharing is not applicable to this article.
